# Impact of fluorine-containing nanoparticle PEGylation on inflammation imaging by ^19^F MRI

**DOI:** 10.1038/s41598-025-29900-8

**Published:** 2025-11-29

**Authors:** Pascal Bouvain, Jonas Schmitz, Matthias Karg, Sebastian Temme, Ulrich Flögel

**Affiliations:** 1 Department of Molecular Cardiology, Experimental Cardiovascular Imaging, Heinrich Heine University, North Rhine-Westphalia, Universitätsstraße 1, 40225 Düsseldorf, Germany; 2 Department of Molecular Cardiology, Heinrich Heine University, North Rhine-Westphalia, Düsseldorf, Germany; 3 Institute of Physical Chemistry 1, Heinrich Heine University, 40225 Düsseldorf, Germany; 4 Institute of Chemistry, Physical Chemistry of Functional Polymers, Martin Luther University Halle-Wittenberg, 06120 Halle (Saale), Germany; 5 Department of Anesthesiology, University Hospital, North Rhine-Westphalia, Düsseldorf, Germany; 6 Cardiovascular Research Institute Düsseldorf (CARID) Heinrich Heine University Düsseldorf, 40225 Düsseldorf, Germany

**Keywords:** FNPs, PEGylation, EPR, ^19^F MRI, Inflammation, Drug delivery, Magnetic resonance imaging

## Abstract

Fluorine-containing nanoparticles (FNPs) are widely used for inflammation imaging by fluorine-19 magnetic resonance imaging (^19^F MRI) due to their biocompatibility and suitability to track immune cells via phagocytic uptake. For targeting approaches beyond passive incorporation, surface PEGylation of FNPs is required to reduce cellular uptake, but is known to prolong blood half-life of the particles. This study investigates the efficacy of FNP PEGylation for inflammation imaging in vivo. FNPs and PEGylated FNPs (^PEG^FNPs) of different size were synthesized and characterized for particle properties and fluorine content. Cellular uptake was explored in CHO, RAW, and J774 cells as well as in whole blood using flow cytometry. For in vivo imaging, a murine lipopolysaccharide (LPS)-induced inflammation model was employed, followed by intravenous injection of FNPs or ^PEG^FNPs and ^19^F MRI to monitor inflammation. PEGylation significantly reduced the uptake of FNPs by macrophages and blood immune cells, as observed through reduced fluorescence and ^19^F signals. Despite reduced cellular uptake in vitro, in vivo ^19^F MRI showed similar signal intensities in inflamed tissues for both FNPs and ^PEG^FNPs, suggesting contributions from both immune cell-associated and non-cell-associated signals for small particles. However, for bigger particles significantly more ^19^F signal was observed in inflamed tissue for FNP compared to ^PEG^FNP. In conclusion, increase in particle size can abolish the non-specific accumulation of FNPs in inflammatory lesions and additionally increase the phagocytosis of FNPs by murine immune cells. This results in a specific immune-cell dependent ^19^F signal with rather no background due to non-specific diffusion.

**Supplementary Information:**

The online version contains supplementary material available at 10.1038/s41598-025-29900-8.

## Introduction

Non-invasive imaging of inflammation by combined ^1^H/^19^F magnetic resonance imaging (MRI) has received increased attention in the past since it can be utilized for visualization of diseases in a variety of preclinical models like graft rejection or myocardial infarction^1,2^. More recently, it has also been used for clinical approaches in a setting of adenocarcinoma patients by labeling dendritic cells and tracking them *in vivo*^3^. ^19^F MRI is based on fluorinated substances and especially perfluorocarbons are used, which are known to be physiologically inert and harmless to cells or organisms^4,5^. However, due to their physicochemical properties (they are neither hydro- nor lipophil but fluorophil) they have to be emulsified into lipids for biological application. The most widely used fluorine-containing nanoparticles (FNPs) are perfluorocarbon nanoemulsions or PLGA nanoparticles which have extensively been used in the past^6–8^. The former one for in vivo applications to track cells and the latter one for ex vivo labeling of immune cells which are afterwards re-implanted and followed over time by combined ^1^H/^19^F MRI.

The general concept of inflammation imaging by FNPs is that upon injection, FNPs are taken up by blood monocytes or other phagocytic cells. These FNP-loaded cells accumulate within inflammatory lesions and can be visualized by combined ^1^H/^19^F MRI^2^. The uptake of FNPs by phagocytic cells was shown in several studies indicating blood monocytes as the main phagocytes of FNPs^2^. However, also neutrophils^9^ or under certain circumstances epicardium-derived stem cells^10^ are able to phagocyte FNPs. Alternatively, they are taken up by local immune cells after extravasation via a leaky endothelium^9^. In contrast to the non-specific passive targeting, a more advanced approach is the active targeting of different cells or structures, like neutrophils^11^ or thrombi^12^ via FNPs. However, a prerequisite for this active targeting is to avoid the non-specific uptake of FNPs by phagocytic cells.

The gold standard to prevent an uptake of FNPs by phagocytic cells is PEGylation of the particle surface i. e. the modification of FNPs with long lipophilic polyethylen-glycol (PEG) chains^13^. These PEG chains introduce a rejective force towards opsonization by serum proteins, which impairs the formation of a protein corona, resulting in a reduced cellular uptake of FNPs by phagocytic cells^14^. Furthermore, the long PEG chains decrease the ζ potential which also minimizes binding of serum proteins to the surface of FNPs^15^. However, most studies investigated the effect of surface PEGylation on cellular uptake in vitro and only less *in vivo*^16–18^. What is known is, that PEGylation of nanoparticles or smaller molecules results in an increased blood half-life enabling the imaging of tumor tissue due to an enhanced permeability and retention effect (EPR)^19^.

The EPR effect was primarily described as a tumor-associated phenomenon^20^. However, it is now well accepted that the described effect is not only tumor but generally inflammation associated^21–23^ as nanoparticles can accumulate within inflamed tissue through leaky endothelium. Because of reduced lymph drainage these particles will stay in this tissue for several days^20,24^. As pointed out above, PEGylation of the nanoparticle surface strongly enhances their half-life in the blood that allows particle diffusion over a prolonged period of time into the tissue via the leaky endothelium and therefore may intensify the EPR effect. Thus, on the one hand, PEGylation diminishes the uptake of nanoparticles by phagocytic cells, which is needed for a proper active targeting. On the other hand, PEGylation leads to elongated blood half-life, which is critical for the EPR effect and it is not clear how the EPR effect influences the inflammation imaging in vivo. Therefore, we systematically investigated the effect of FNP PEGylation on immune cell uptake and ^19^F signal deposition in vitro and in vivo.

## Results

### PEGylation decreases the cellular uptake of FNPs by cultured cell lines

**Fig. 1:**
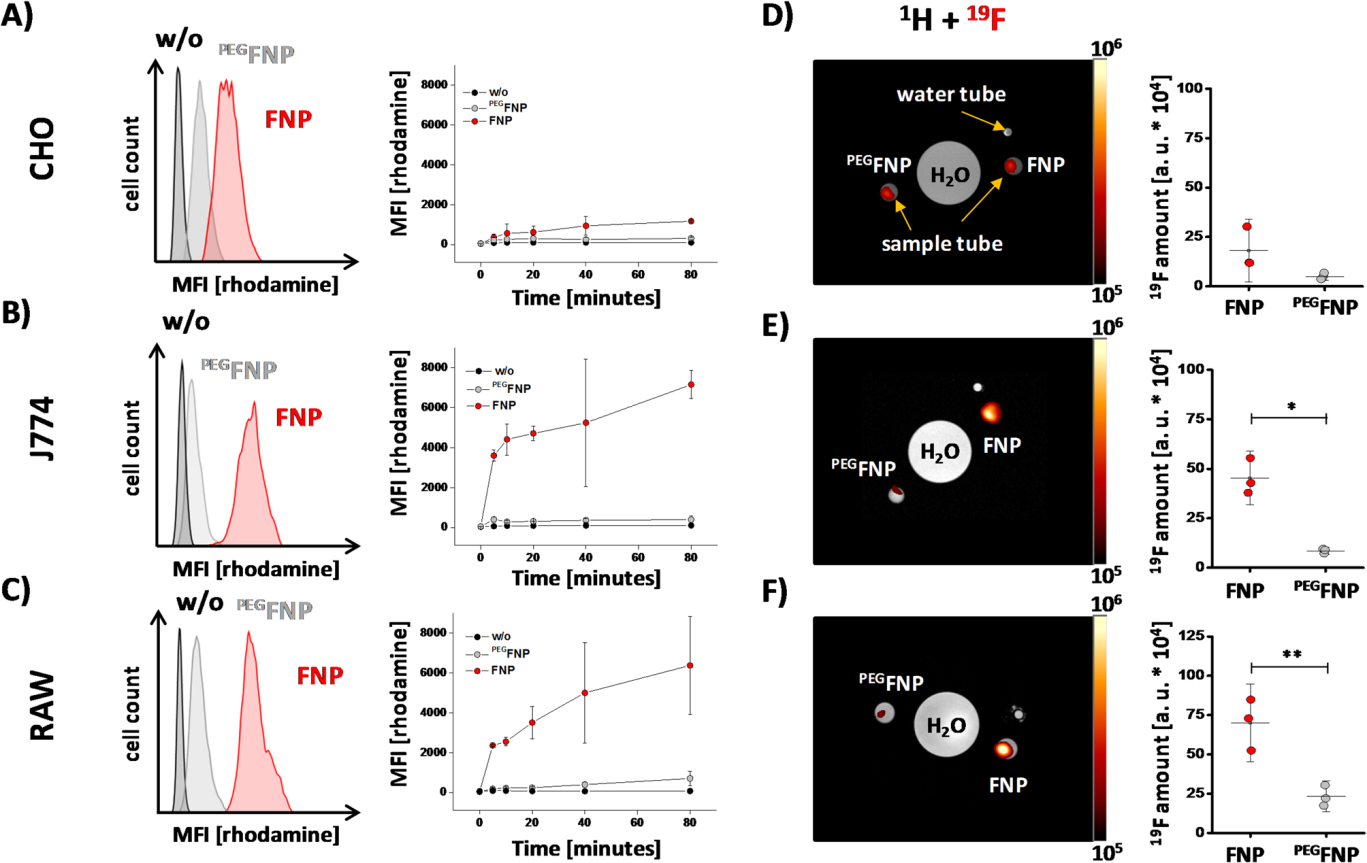
Analysis of FNP and ^PEG^FNP labeling by different cell lines. CHO (**A**) and the macrophage cell lines (J774 (**B**) and RAW (**C**)) were incubated with rhodamine-labelled FNPs (red) or ^PEG^FNPs (grey) or were left untreated (black) at 37 °C over 80 min. At different time points, rhodamine fluorescent intensities were measured by flow cytometry. The histogram plots represent the measurements after 80 min of incubation. The CHO (**D**), macrophage cell lines (J774 (**E**) and RAW (**F**)) were incubated with FNPs (red) or ^PEG^FNPs (grey) at 37 °C for 24 h. After several washing steps the cells were transferred into PCR tubes and the amount of ^19^F within the cell pellet was determined by ^19^F MRI. On the left, axial ^1^H MR images of a buffered filled 2 ml reaction tube as well as the PCR tubes are depicted. The hot iron overlay shows the amount of fluorine within the cell pellets. Quantitative analysis for the different cell lines are depicted on the right. Data points represent mean ± SD of n = 3 (A–F) independent experiments. * = *p* < 0.05; ** = *p* < 0.01 verified by student´s *t*-test.

To investigate the impact of FNP surface PEGylation on cellular uptake, we incubated non-phagocytic CHO (chinese hamster ovarian) cells as well as the macrophage cell lines J774 and RAW over a time period of 80 min with rhodamine-labelled FNPs or ^PEG^FNPs at 37 °C and analyzed the uptake by flow cytometry. Samples were withdrawn before (0 min) or at several time points after addition of FNPs/^PEG^FNPs (5, 10, 20, 40 and 80 min). We observed that labelling CHO cells with FNPs led to only very low levels of rhodamine fluorescence signal which slightly increases over time (Fig. 1A). Nevertheless, incubation with ^PEG^FNPs resulted in a 60% reduction of the cell-associated fluorescence signal in CHO cells after 80 min. As expected, J774 macrophages showed strong fluorescence signals already after 5 min which increased over the observation time of 80 min (Fig. 1B). Treatment of J774 cells with ^PEG^FNPs nearly completely abolished (over 90% reduction) the rhodamine fluorescence of the J774 cells. Similar results were observed for RAW macrophages, but with the slight difference that RAW cells had a lower level of fluorescence signal in the early phase and the cell-associated rhodamine signal increased more gradually over time compared to J774 macrophages (Fig. 1C). Histograms of all three cell lines show an overlay of cells incubated with FNPs (red) and ^PEG^FNPs (grey) after 80 min of incubation (Fig. 1A–C).

Next, we investigated if the decreased cellular uptake of ^PEG^FNPs is also detectable by ^1^H/^19^F MRI. Therefore, CHO, J774 and RAW were incubated with FNPs or ^PEG^FNPs over a period of 24 h. After washing, cells were fixed with PFA, transferred into reaction tubes and pelleted by centrifugation. Afterwards, the ^19^F content of the cell pellet was determined by combined ^1^H/^19^F MRI. Figure 1D–F shows axial MR slices of the tubes containing the cell pellets as well as the corresponding hot iron ^19^F signal merged with the grey ^1^H data. In concordance with the flow cytometric data, CHO cells showed only low ^19^F signals for FNPs and even less when incubated with ^PEG^FNPs (Fig. 1D, right). In contrast, J774 and RAW macrophages displayed a high ^19^F content after incubation with FNPs and significantly less fluorine signals after treatment with ^PEG^FNPs (Fig. 1E + F, right).

### PEGylation decreases the cellular uptake of FNPs in whole blood

**Fig. 2:**
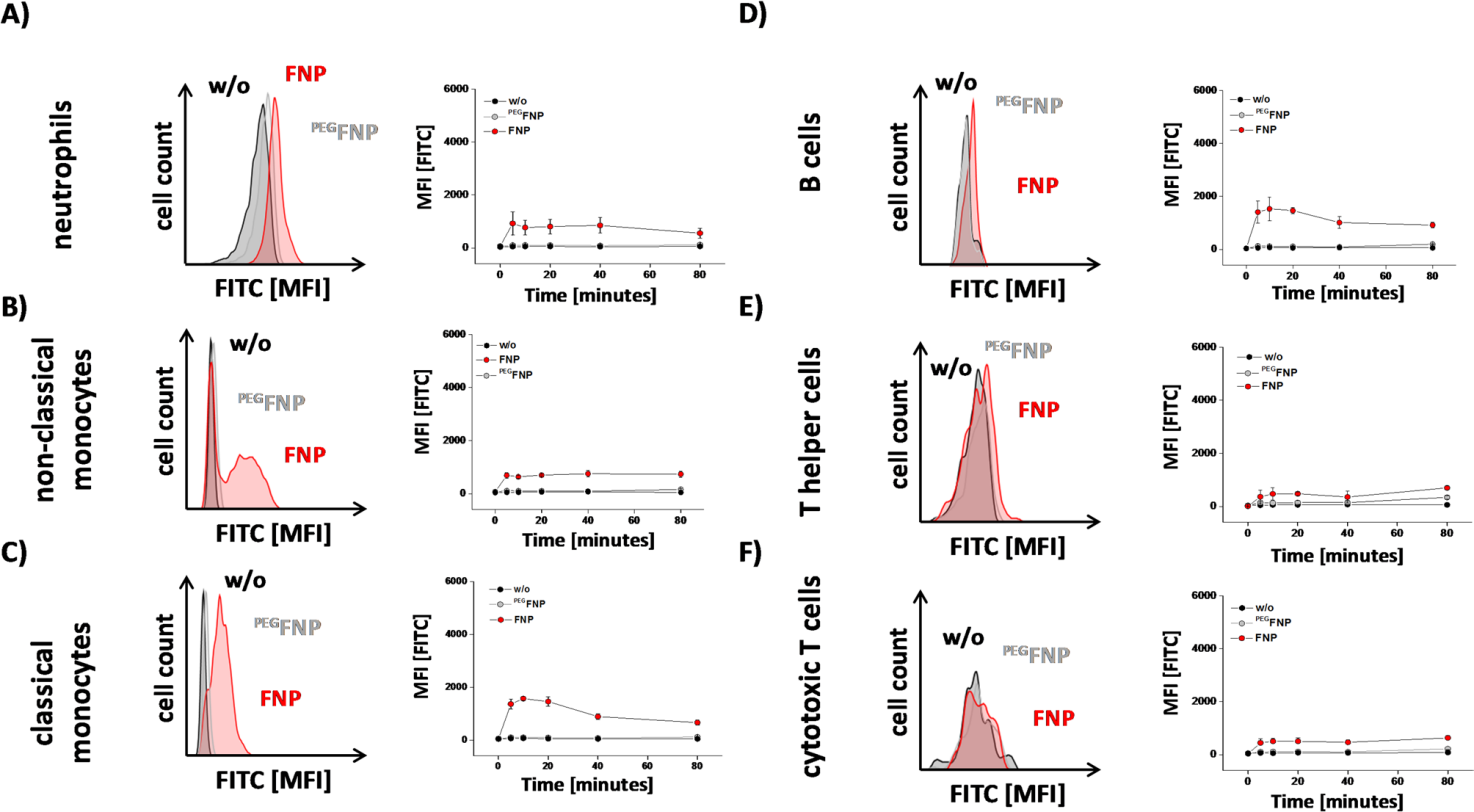
Cellular uptake of FNPs and ^PEG^FNPs by murine immune cells. Binding of FNPs (red) and ^PEG^FNPs (grey) by isolated murine immune cells in whole blood. As a ctrl, immune cells were left untreated (black). Immune cells were incubated over 80 min in whole blood with FNPs or ^PEG^FNPs, isolated and the fluorescence intensities were determined by flow cytometric analysis for neutrophils (**A**), non-classical monocytes (**B**) classical monocytes (**C**), B cells (**D**) T helper cells (**E**) and cytotoxic T cells (**F**). Data points represent mean ± SD of n = 4–6 independent experiments.

An important factor for the uptake of FNPs in vivo are serum proteins from the blood^25^. Upon injection into the blood, a shell of serum proteins generates a so called protein corona, which strongly impacts the uptake by phagocytic cells^26^. Therefore, we investigated the uptake of FNPs and ^PEG^FNPs into murine immune cells in whole blood (Fig. 2). Upon incubation of the cells with FNPs and withdrawal of samples before (0 min) and at distinct time points after incubation (5, 10, 20, 40, 80 min) the cells were analyzed by flow cytometry. Samples were labelled with mAbs to differentiate neutrophils, classical and non-classical monocytes (myeloid cells) as well as T- and B-cells (lymphoid cells). We observed an increasing rhodamine fluorescence signal over 80 min for neutrophils and both monocyte subsets (Fig. 2A–C). Among all myeloid cell-types, classical monocytes displayed the strongest fluorescence signals for FNPs (Fig. 2C). PEGylation of FNPs significantly reduced the fluorescence signals for all three cell types over time (grey, Fig. 2A–C). In contrast to the myeloid cells, the lymphoid cells showed only low (B-cells) or apparently no uptake of FNPs (CD4 + and CD8 + T-cells). Incubation with ^PEG^FNPs resulted in a decreased fluorescence signal for B-cells almost at the same level as the rhodamine signal in T-cells (Fig. 2D–F).

### Physicochemical characterization of FNPs and PEGylated-FNPs

**Fig. 3:**
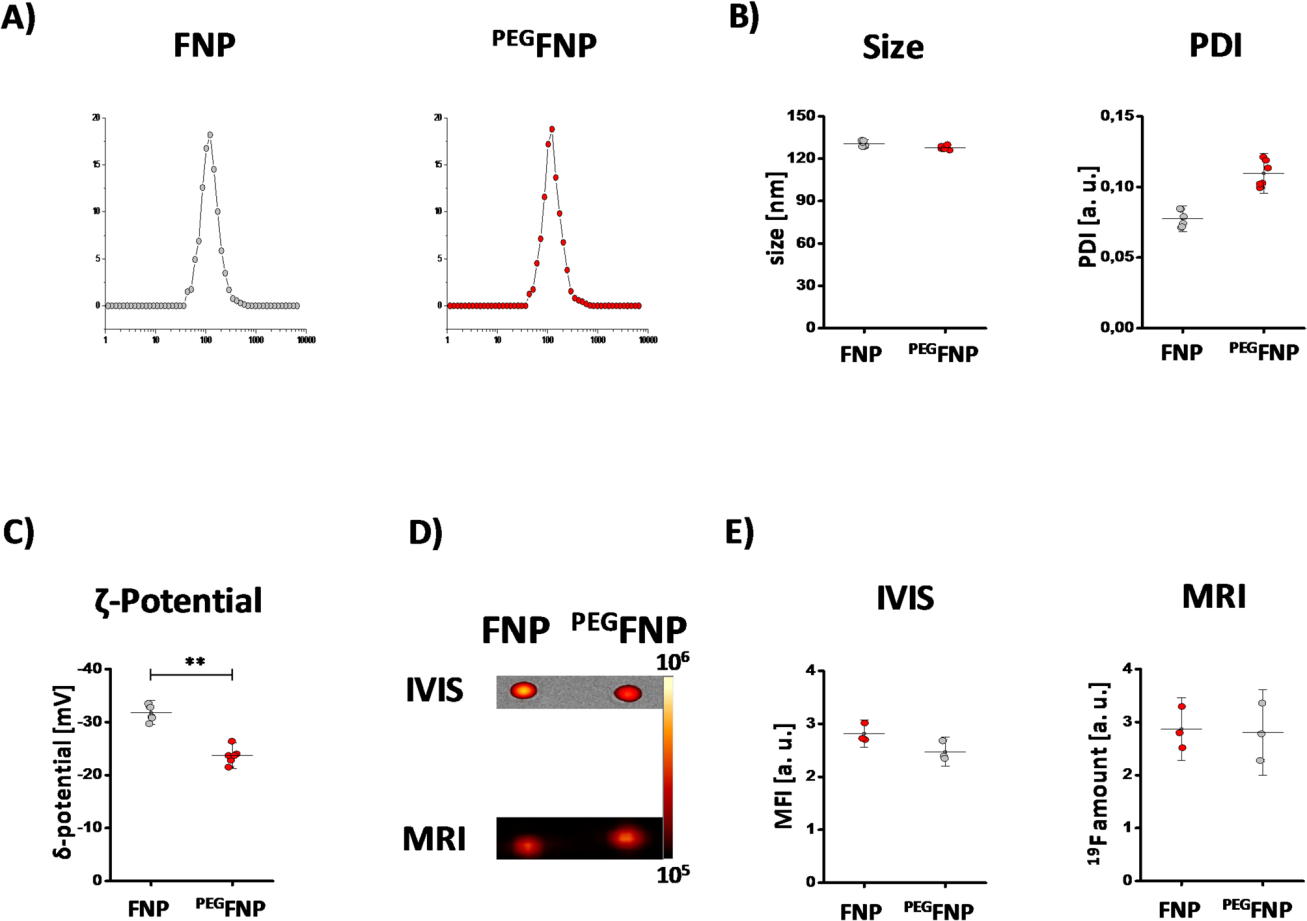
Characterization of FNPs and ^PEG^FNPs. (**A**–**C**) FNPs (red) and ^PEG^FNPs (grey) were analyzed by dynamic light scattering regarding their size (A), size distribution (PDI) (B) and the ζ-potential (C), indicating a higher PDI value and lower ζ-potential for PEGylated FNPs. (**D**–**E**) To determine the mean fluorescence intensities of FNPs and ^PEG^FNPs the rhodamine fluorescence was measured via an *in-vivo* imaging system indicating a small but not significant reduction in mean fluorescence for the ^PEG^FNPs. To examine the fluorine content, FNPs and ^PEG^FNPs were analyzed by ^1^H/^19^F MRI. Data are mean values ± SD of n = 3 independent experiments. ** = *p* < 0.01 verified by student´s *t*-test.

To exclude that alterations in the uptake are associated with physicochemical differences in the different FNPs, we characterized FNPs and ^PEG^FNPs by dynamic light scattering (DLS), fluorescence imaging as well as ^19^F MRI to determine the size, polydispersity index (PDI), ζ potential, the rhodamine fluorescence signal as well as the ^19^F content (Fig. 3). DLS analysis revealed that the size of the nanoemulsion droplets is approximately 130 nm and does not significantly differ between FNPs and ^PEG^FNPs (FNPs: 130.63 nm ± 1.78 nm; ^PEG^FNPs: 127.68 nm ± 1.27 nm) (Fig. 3A). The PDI was in the range of 0.1 for both FNPs and ^PEG^FNPs (FNPs: 0.077 ± 0.005; ^PEG^FNPs: 0.11 ± 0.0086) indicating a monodisperse distribution for both preparations (Fig. 3B). As expected, the ζ potential of ^PEG^FNPs was less negative compared to FNPs (FNPs: -31.78 mV ± 1.36 mV; ^PEG^FNPs: -23 mV ± 1.47 mV) (Fig. 3C).

Next, we also analyzed the fluorescence signal by an in vivo imaging system (IVIS) and the ^19^F content by ^19^F MRI (Fig. 3D + E). IVIS measurements revealed a slight reduction in the mean fluorescence signal for PEGylated FNPs compared to FNPs (MFI [a. u.] = FNPs: 2.81 ± 0.17; ^PEG^FNPs = 2.47 ± 0.18), that was statistically not significant. However, no differences in signal intensity were detectable for the ^19^F measurements (^19^F amount [a. u.]: FNPs = 2.86 ± 0.39; ^PEG^FNPs = 2.8 ± 0.54). Furthermore, we investigated the long-term stability of the nanoemulsion. On one hand we characterized them after different time points (1, 2, 3, 4 weeks) of storage at 4 °C via DLS measurements and additionally cryo-TEM analysis. On the other hand, we analyzed their stability at 37 °C over 24 h. In both cases we could not detect strong impact on their physicochemical features due to the storage conditions (see suppl Fig S1).

### In vivo ^***19***^***F deposition is not changed by FNP PEGylation***

**Fig. 4:**
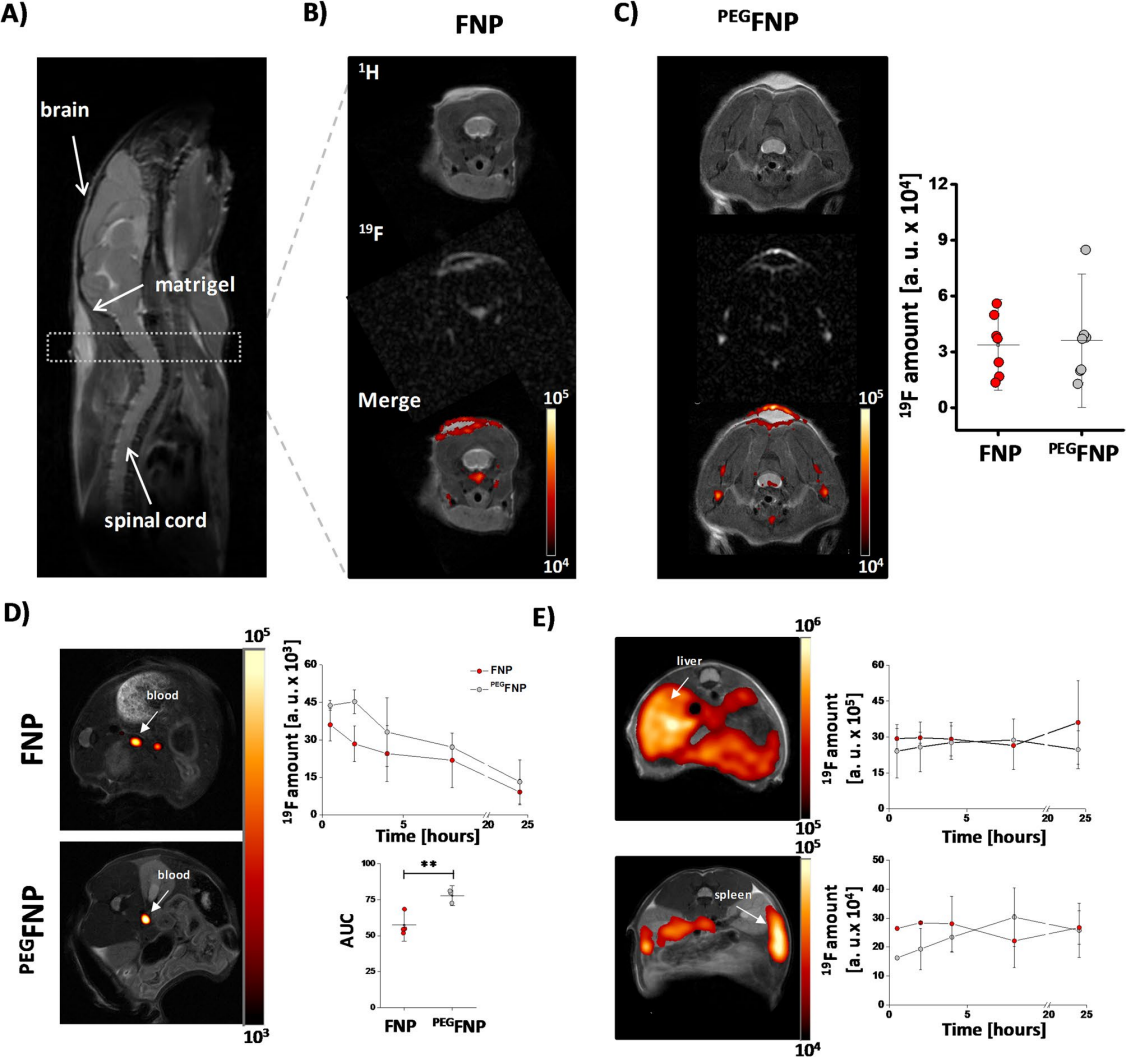
No differences between FNPs and ^PEG^FNPs for in vivo ^19^F inflammation imaging. (**A**) Matrigel, mixed with LPS, were implanted into the neck of mice to induce a local inflammation. The ^1^H MR image shows the orientation of the matrigel on top of the neck of the mouse. (**B** + **C**) First row represents one axial scan through the matrigel of the FNP (B) or ^PEG^FNP (C) treated animals. In the middle, the corresponding ^19^F MR image is depicted. A merge of the ^1^H and ^19^F MR images is shown below. Quantification on the right shows the in vivo ^19^F signals of the matrigels. (**D** + **E**) To determine the blood half time and the wash-in in liver and spleen the FNPs/^PEG^FNPs were injected i. v. and after distinct time points the ^19^F amount was measured within the *vena cava*, the liver and the spleen. Data are mean values ± SD of n = 7 (B + C) or n = 3–4 (D) independent experiments. ** = *p* < 0.01 verified by student´s *t*-test.

To verify whether the reduced FNP uptake by immune cells upon PEGylation is also reflected by decreased ^19^F signals in inflamed tissue, we used a subcutaneous inflammation model induced by implantation of LPS-doped matrigel into the neck of mice^9^. Matrigel is fluid at 4 °C but converts into an almost solid gel at body temperature, while addition of LPS leads to a localized immune response with strong infiltration of neutrophils into the matrigel plug starting already within the first hours after implantation^9^. One day after matrigel implantation FNPs or ^PEG^FNPs were intravenously injected and after further 24 h mice were subjected to noninvasive in vivo ^1^H/^19^F MRI to determine the ^19^F signal within the matrigel plug. First, anatomical ^1^H MR images in sagittal orientation were recorded to localize the implanted plug, which is visible as bright structure in the ^1^H image (Fig. 4A). Afterwards, anatomically corresponding ^19^F MR images were acquired to cover the whole area of the plug (Fig. 4B). Subsequently, ^19^F data were merged with the ^1^H images to identify the anatomical location of the ^19^F signals. The upper part of Fig. 4B + C shows axial ^1^H images of the neck area with the bright matrigel. In the middle part, the ^19^F data with the same geometrical orientation as above is displayed. Merging of ^1^H and ^19^F data demonstrates that the ^19^F signal is mainly localized at the border area of the matrigel plug (Fig. 4B/C, lower part). Interestingly, quantification of the ^19^F data revealed comparable matrigel-associated ^19^F signals 24 h after injection of FNPs or ^PEG^FNPs (Fig. 4C) despite the diminished uptake of ^PEG^FNPs by neutrophils and monocytes observed both in vitro and in vivo (see above).

To investigate if there are any accompanying differences in blood half-life or accumulation in the reticuloendothelial system, we investigated the ^19^F signal in the blood circulation as well as the wash-in of FNPs/^PEG^FNPs into the liver and spleen over a time period of 24 h after intravenous application. Figure 4D + E shows the quantitative analysis of the ^19^F signals in the blood (left, upper part), the liver (right, upper part) and the spleen (right, lower part). As expected, we detected much longer retention times for ^PEG^FNPs with a significantly higher ^19^F signal in the blood over the entire observation period of 24 h (Fig. 4D, lower part). For liver and spleen, in the early phase ^19^F signal deposition tended to be lower for ^PEG^FNPs as compared to FNPs, which however gradually aligned over the observation period (Fig. 4E).

### *PEGylation impairs cellular uptake of FNPs *in vivo

**Fig. 5:**
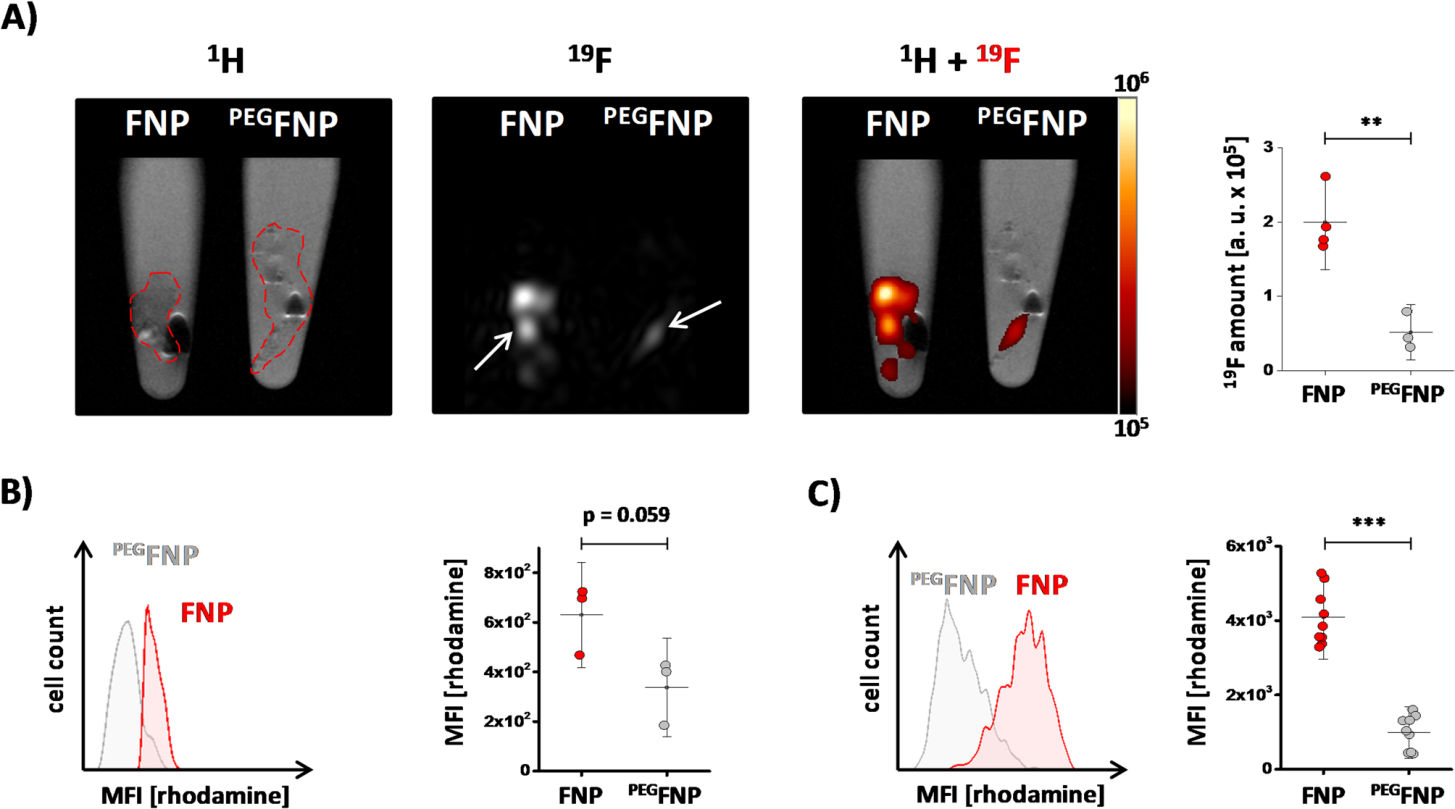
PEGylation impairs labeling by FNPs in ex vivo measured inflammatory hot spots. (**A**) Matrigel was subcutaneously implanted into the neck of mice to induce a local inflammation. One day after implantation FNPs or ^PEG^FNPs were injected intravenously and again one day after injection the matrigels were excised, washed intensively and fixed with PFA. Afterwards they were analyzed by MRI to determine the ^19^F signal within the matrigel. On the left, the sagittal ^1^H overview MR images of the matrigel area are shown (matrigels are highlighted via red circles). The matrigel can be seen as dark grey structure (red circles). The middle row indicates the corresponding ^19^F MR images. The overlay of ^1^H and ^19^F MR images show that the ^19^F signal is associated with the matrigel. (**B**) The FNPs or ^PEG^FNPs were injected 24 h after matrigel implantation and two hours later neutrophils were isolated from the matrigel plug to determine the rhodamine labeling by flow cytometry. (**C**) Neutrophils from the matrigel plug were isolated and incubated ex vivo with FNPs or ^PEG^FNPs and the rhodamine fluorescence was determined by flow cytometry. Histogram plot on the left shows a representative example of the rhodamine signal from FNP (red) and ^PEG^FNP (grey) treated neutrophils. Data are mean values ± SD of n = 4, 3 (A), n = 3 (B) and n = 9 (C) independent experiments. ** = *p* < 0.01; *** = *p* < 0.001 verified by student´s *t*-test.

Since we could not detect any differences between ^PEG^FNPs and FNPs in the in vivo ^19^F inflammation imaging experiments, we next tried to separate the ^19^F signal from infiltrated immune cells and any signal belonging to free FNPs within the matrigel. To this end, one day after matrigel implantation FNPs or ^PEG^FNPs were intravenously injected and after further 24 h the plug was carefully excised, washed with PBS, fixed with PFA and finally subjected to ^1^H/^19^F MRI (Fig. 5A). Sagittal T2-weighted ^1^H measurements were performed to display the excised matrigel plugs inside PBS-filled reaction tubes (Fig. 5A, left) and afterwards, the ^19^F signal was determined from the same field of view (Fig. 5A, middle). Merging of the ^1^H and ^19^F datasets revealed that the localization of the ^19^F signals corresponds to the matrigel plugs (Fig. 5A, right). Quantitative analysis of the total ^19^F signal revealed a significantly stronger ^19^F signal for matrigel plugs isolated from mice which received FNPs compared to mice treated with ^PEG^FNPs (Fig. 5A).

To further scrutinize if this difference in ^19^F signal is based on an altered cellular association of FNPs and ^PEG^FNPs, the same protocol as above was applied (FNPs/^PEG^FNP injection one day after plug implantation) and two hrs later neutrophils were isolated from the matrigel. Subsequent flow cytometry revealed higher rhodamine fluorescence signals for neutrophils isolated from mice treated with FNPs compared to ^PEG^FNPs (Fig. 5B, left). Quantification of the fluorescence intensity did not reach the level of significance, maybe due to degradation of the fluorochrome^27^, but showed a clear trend to reduced values for ^PEG^FNPs (Fig. 5B, right). Furthermore, we isolated matrigel-infiltrated cells, predominantly neutrophils, and incubated them ex vivo with FNPs or ^PEG^FNPs and analyzed their uptake by flow cytometry. Figure 5C left shows a histogram of the flow cytometric data and quantification of the mean fluorescence intensity again revealed a significant reduction in the rhodamine signal for ^PEG^FNPs as compared to FNPs (Fig. 5C, right).

### Increase in size can diminish EPR-dependent accumulation of FNPs

**Fig. 6:**
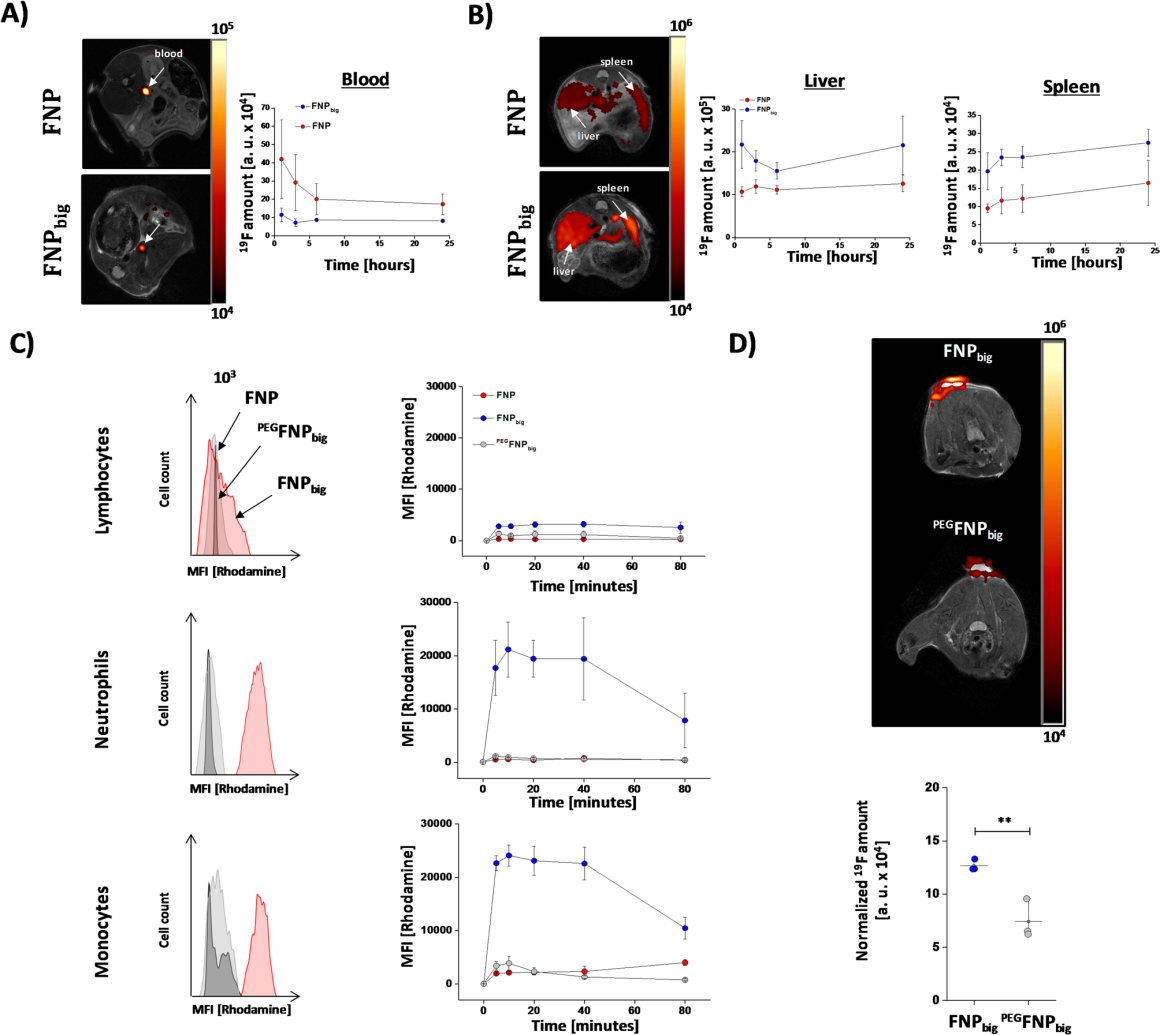
Increase in size can abolish EPR-dependent accumulation of FNPs. (**A **+** B**) To determine the blood half time and the wash-in in liver and spleen the FNP/FNP_big_ were injected i. v. and after distinct time points the ^19^F amount was measured within the *vena cava*, the liver and the spleen. (**C**) Murine immune cells were isolated from the blood and afterwards incubated with FNP, FNP_big_ or ^PEG^FNP_big_ over 80 min. At distinct time points the uptake into lymphocytes, monocytes and neutrophils was determined via flow cytometric analysis. (**D**) FNP_big_ or ^PEG^FNP_big_ were injected intravenously into the tail vein of mice 24 h after implantation of matrigel mixed with LPS into the neck of mice. Again, 24 h after injection the ^19^F signals within the matrigel were determined via combined ^1^H/^19^F MRI. The signal was normalized to the area of the matrigel. Data are mean values ± SD of n = 3 (A), n = 3 (B), n = 3 (C) and n = 3 (D) independent experiments. ** = *p* < 0.01 verified by student´s *t*-test.

So far, the results indicate that PEGylation indeed abolishes uptake of FNPs in vivo, but that the EPR effect can lead to non-specific accumulation of FNPs within inflammatory lesions over time. To minimize this passive accumulation through the leaky endothelium and to reduce circulation time, we generated bigger particles of roughly 300 nm (FNPs_big_; suppl. Fig. S2). The increase in particle size demonstrated a clearly enhanced uptake by different cell culture cells, especially by macrophage cell lines (see suppl. Fig. S2). Analysis of biodistribution and blood half-time demonstrated a faster wash out from the blood already 1 h after injection (Fig. 6A) accompanied by stronger accumulation in liver and spleen as compared to normal FNPs (Fig. 6B). Furthermore, FNPs_big_ show an even increased uptake not only by different cell culture cells (suppl. Fig. S2) but also by murine immune cells, especially monocytes and neutrophils (Fig. 6C), while a PEGylation of FNPs_big_ (^PEG^FNPs_big_) completely abolishes these effects. Of course, FNP_big_ were also characterized for their stability due to different storage conditions (see suppl. Fig. S3). Finally, we injected FNPs_big_ or ^PEG^FNPs_big_ into mice which have received an LPS/matrigel implantation. ^19^F MR measurements on the following day indeed revealed a significant weaker signal for ^PEG^FNPs_big_ in comparison to FNPs_big_ (Fig. 6D). Obviously, the combined effect of a reduced blood half-life of ^PEG^FNPs_big_ and an even stronger immune cell uptake of FNPs_big_ largely improves the specificity of FNPs for selective inflammation imaging and diminishes their EPR-related accumulation in inflamed foci.

## Discussion

In the present study we investigated the impact of FNP PEGylation on inflammation imaging by ^19^F MRI and demonstrated the functionality of a PEGylation to impair the uptake of FNPs in vitro into cell culture lines as well as *ex* and in vivo in murine immune cells via flow cytometry and ^19^F MRI. Controversially to the cell-associated ^19^F signal, we found no differences in the ^19^F signal within a LPS-based matrigel inflammation model in vivo but significant differences in ex vivo measurements of the dissected and washed matrigels. Additionally, neutrophils show an increased uptake of FNPs in the circulation and also within the inflammatory hot spot compared to PEGylated FNPs. This indicates that ^PEG^FNPs can accumulate within inflammatory lesions due to the enhanced permeability and retention effect. However, increasing the particle size can overcome this limitation by reducing blood half-time of FNPs and increasing their uptake by immune cells to reduce passive accumulation of free FNPs.

‘Neat’ FNPs are commonly used as contrast agents to visualize immune cells by non-invasive ^19^F MRI^2,9^. Several applications of FNPs have been described, e. g. for cell tracking via *ex vivo*^28^ but also in situ labeling^29^ and even active targeting approaches against structures beyond phagocytic cells, such as thrombi^11,12^. For the latter, it is crucial to minimize the phagocytotic uptake of the targeted contrast cargo by the reticuloendothelial system (RES). The gold standard for generating a so-called stealth effect of nanoparticles is the introduction of poly-ethylene–glycol (PEG) into the particle surface, a process which is called PEGylation^30^. Although there are several reports dealing with PEGylation, the data availability concerning FNPs is rudimentary – most investigations up to now were carried out with liposomes^31–33^. Here, we could show that in vitro FNP/^PEG^FNP-treated cell culture cells (CHO, RAW, J774) as well as ex vivo whole blood incubated murine immune cells indeed show a strong decrease in fluorescence and ^19^F signal due to surface PEGylation of FNPs. A reduced uptake of PLGA particles due to surface PEGylation has already been shown in vitro for J774 macrophages^16^, even in serum-containing media^17^. Similar effects were seen for FNPs, where PEGylation impairs the uptake into isolated human monocytes^18^ which is in line with our findings.

Upon intravenous injection of FNPs or ^PEG^FNPs, we could not see any differences in the fluorine signal between both emulsions within a LPS-based matrigel inflammation model. In contrast, after dissection of the matrigel-plug and ex vivo scanning, there is significantly more ^19^F signal in the FNP-treated group compared to the ^PEG^FNP treated. A possible explanation is, that FNPs are taken up by immune cells from the circulation or local immune cells, which leads to a cell-dependent ^19^F signal within the matrigel. In contrast, PEGylation leads to signals due to EPR-dependent diffusion of ^PEG^FNPs into the inflamed area without or only few uptake by the local immune cell environment. These results are also in accordance with flow cytometric analysis of neutrophils isolated from the matrigel upon injection, showing stronger fluorescence signals for FNPs compared to ^PEG^FNPs. This effect is even more pronounced when isolated neutrophils from the matrigel are incubated ex vivo with the particles.

We furthermore could show that this EPR-dependent diffusion can be avoided by increasing the size of FNPs to roughly 300 nm. This leads to (i) less circulation time and finally less EPR-dependent accumulation of free FNPs within leaky endothelium and (ii) increased uptake by murine immune cells which further increases the cell-dependent ^19^F signal. It is well known that bigger particles have a faster wash out from the blood^34^ and also an increased uptake by immune cells since size above 250 nm activates phagocytosis^35^. This combination results in a specific immune cell dependent signal within the matrigel with rather no background noise via EPR-dependent diffusion. However, an increase in particle size can also lead to unwanted activation of the cells. For imaging, this must of course be avoided and the cells should remain in their normal state.

Although flow cytometric and ^19^F MRI data are in reasonable accordance in this study, it has to be taken into account that localization of the fluorescent dye and ^19^F signal may drift apart over time. The fluorescent dye is bound either to the phospholipids or the targeting ligand located at the surface of the FNPs while the flourine-containing compounds are trapped in the core of the particles. After cellular uptake and internalization of the FNPs, the lipid layer might be metabolized and/or integrated in other membranous structures and the binding ligand may still stick at its target structure, which leads to a separation of the fluorescence and the ^19^F label. However, these limitations apply also for almost all over kinds of dual-mode nanoparticle and should considered when interpreting data from long-term experiments.

In summary, we demonstrate that ^PEG^FNPs can accumulate within the inflamed area because of the enhanced permeability and retention effect. FNPs will be taken up by the local immune cells, in this case neutrophils, as well as immune cells from the circulation. However, PEGylation impairs the uptake and so ^PEG^FNPs can stay in the extracellular space. Increase in size can furthermore reduce the blood half-life in combination with boosted phagocytosis to avoid this non-specific accumulation of FNPs within inflamed tissue and to result in a more specific immune cell dependent signal. In the future, this work may contribute to further improving the accuracy of molecular imaging of immune cells by enhancing the ^19^F signal strength. The significantly increased uptake would also allow for the use of smaller amounts of FNPs, thereby minimizing the non-specific signal in the liver and spleen.

## Methods

### Preparation and Characterization of fluorinated nanoparticles formation

Fluorinated nanoparticles (FNPs) were prepared as previously reported^2,36^. In brief, 2.4% (for “normal” FNPs) or 0.24% (for “bigger” FNPs) (w/w) phospholipid (Lipoid S75, Lipoid AG, Ludwigshafen, Germany) was dissolved in 10 mM phosphate-glycerol buffer (7 mM Na_2_HPO_4_, 3 mM NaH_2_PO_4_, 2.5% glycerol, pH 7.4) and pre-emulsified for 30 min. Next, 20% (w/w) perfluoro-15-crown-5-ether (ABCR, Karlsruhe, Germany) was added to the dispersion and a crude emulsion was formed by high shear mixing (Ultra Turrax TP 18/10; IKA-Werke, Staufen, Germany). High shear homogenization was performed in 5 cycles at 1000 bar using LV1 microfluidizer (Microfluidics Corp, Westwood, MA, USA).

To generate fluorescent and PEGylated FNPs, 0.01 mol% rhodamine labelled lipids (Rhodamine-DHPE, Thermo-Fisher, Waltham, MA, USA) and 5 mol% PEGylated lipids (DSPE-PEG_2000_, Lipoid) were additionally added to the lipoid S75 mixture, a crude emulsion was formed by high shear mixing and the nanoemulsions were prepared by high shear homogenization in 5 cycles at 1000 bar using LV1 microfluidizer.

For all ex vivo experiments, cells were incubated with 10 µl FNPs/ml which corresponds to ≈80 mmol ^19^F nuclei per ml or with 50 µl FNPs/ml which corresponds to ≈200 mmol ^19^F nuclei per ml. For in vivo experiments, mice received a body weight (BW) adapted injection of FNPs with 3 mmol PFCE/kg BW (→ ≈65 mmol ^19^F nuclei per kg BW) via the tail vein.

### Characterization of FNPs

FNP, ^PEG^FNP, FNP_big_ and ^PEG^FNP_big_ were characterized by dynamic light scattering (DLS) on a Nanotrac instrument (Microtrac, Krefeld, Germany) to determine the hydrodynamic diameter, the polydispersity index (PDI) and the ζ-potential. To this end, FNPs were diluted 1:100 in H_2_O and measured for 30 s each measurement and 10 repeats. Furthermore, we analyzed the fluorescence via an IVIS Lumina II imaging system (Perkin Elmer, Rodgau, Germany) and the ^19^F amount by MRI as described below. To measure the fluorescence, 10 µl of FNPs were spotted on a glass plate, while analysis of the ^19^F content was conducted with 10 µl FNPs in a PCR tube.

### Cryo-TEM analysis

FNPs were diluted with sample buffer to minimize particle aggregation and therefore enable proper size measurements, which were carried out as previously described^37^.

### Cell culture assays

For all cell culture experiments, we used Chinese hamster ovarian cells (CHO; ECACC 85,050,302), RAW264.7 macrophages (ECACC 91,062,702) or J774.2 macrophages (ECACC 85,011,428). Cells were cultivated in DMEM high glucose (Merck KGaA, Darmstadt, Germany) with 10% FBS (Biochrom, Berlin, Germany), 1% penicillin/streptomycin (Biochrom, Berlin, Germany), 1% glutamax (Sigma-Aldrich, Darmstadt, Germany) and 1% sodium pyruvate (Sigma-Aldrich, Darmstadt, Germany) in a CO_2_ incubator (5%) at 37 °C in a water saturated atmosphere. Cells were split at 80% confluency and medium was replaced every 3 days.

(a) cellular uptake of FNPs/^PEG^FNPs determined by flow cytometry: To determine the cellular association of FNPs and ^PEG^FNPs, 1 × 10^6^ cells were transferred into a 15-ml falcon, resuspended in 1 ml DMEM cell culture medium and 10 µl of the FNPs or ^PEG^FNPs were added. Cells were incubated at 37 °C on a MACSmix™ tube rotator (Miltenyi Biotec, Bergisch Gladbach, Germany). At distinct time points (0, 5, 10, 20, 40, 80 min), 50 µl of the cell suspension were transferred into 2 ml ice-cold PBS to stop the reaction. Cells were washed with PBS and resuspended in MACS buffer (PBS, 2% BSA, 1 mM EDTA) with 1 µg/ml DAPI (Merck). Cells were analyzed by flow cytometry using LSRfortessa flow cytometer (BD Biosciences, Germany, Heidelberg). To separate the cells from debris, we utilized the forward and side scatter profile for size and granularity. In the next step, non-viable cells were identified by DAPI staining (DAPI = 4′,6-diamidin-2-phenylindol). DAPI-positive cells were excluded from the analysis, since these cells do not perform active endocytosis. To determine the amount of the fluorescence signal derived from FNP uptake, we calculated the mean fluorescence intensity of the DAPI-negative cell population.

(b) cellular uptake of FNPs/^PEG^FNPs determined by ^19^F MRI: To determine the uptake of FNPs or ^PEG^FNPs by ^19^F MRI, 1 × 10^6^ cells were covered with 2 ml of fresh cell culture medium. Afterwards 50 µl of FNPs were added and the cells were incubated for 24 h at 37 °C. On the next day, the cells were washed five times with 2 ml PBS and detached with 1 ml Trypsin/EDTA (Gibco, Rockford, IL, USA) at 37 °C for 10 min. 3 × 10^6^ cells were pooled, pelleted by centrifugation and fixed in 4% PFA (paraformaldehyde) solution for 10 min, washed with PBS, transferred into a PCR tube and pelleted again by centrifugation.

### Animal experiments

Animal experiments were approved by the institutional review board “Landesamt für Natur, Umwelt und Verbraucherschutz Nordrhein-Westfalen” and were performed in accordance with the national guidelines on animal care Az: 81–02.04.2018.A468. Male mice (C57BL/6; 20–30 g BW (body weight); 10–12 weeks of age) used in this study were obtained from Janvier (Le Genest-Saint-Isle, France), housed at the central animal facility of the Heinrich Heine University (Düsseldorf, Germany) and fed with a standard chow diet and received tap water ad libitum. The study was performed following the ARRIVE (Animal Research: Reporting of In Vivo Experiments) guidelines 2.0. All methods were performed in accordance with the relevant guidelines and regulations.

#### Immune cell isolation from murine blood

To obtain circulating immune cells, heparinized blood was withdrawn by venous puncture of the inferior vena cava. Blood was collected via a 23G cannula in heparin-aerated collection tubes. Erythrocytes were lysed by adding the fourfold volume of ammonium chloride buffer (pH 7.4). After 10 min of incubation at room temperature the samples were centrifuged at 350 × g for 10 min at 20 °C.

#### Matrigel/LPS inflammation model

For implantation of matrigel plugs, animals were anesthetized with isoflurane, placed on a 37 °C warming plate and the neck was shaved. Subsequently, 50 µl of a fluid matrigel solution (BD Biosciences, Heidelberg, Germany) was injected subcutaneously into the neck using a 0.5-ml microsyringe (BD Biosciences; Heidelberg, Germany). To induce local inflammation, 50 µg of lipopolysaccharide (LPS salmonella typhimurium, Calbiochem, Darmstadt, Germany) was added to the matrigel prior to the injection. The matrigel is fluid at 4 °C but becomes a solid gel at body temperature. After 24 h, a single bolus of FNPs, ^PEG^FNPs, FNP_big_ or ^PEG^FNP_big_ were injected via the tail vein. To examine the ^19^F signal in matrigel, mice were anesthetized by 5 vol% isoflurane and anesthesia was maintained by 1.5 vol% isoflurane on a warming plate at 37 °C. Matrigel was isolated 24 h after injection of FNPs. The mice were killed via cervical dislocation and the matrigel, visible as a white spot, was carefully excised from the skin, washed several times in PBS and fixed with 200 µl of 4% PFA.

#### Biodistribution of fluorinated particles

To determine the biodistribution of FNPs, ^PEG^FNPs, FNP_big_ or ^PEG^FNP_big_, 3 mM per Kg BW were injected intravenously and ^19^F intensities were determined in blood, liver and spleen at distinct time points after injection.

### ^***1***^***H/***^***19***^***F magnetic resonance imaging***

Experiments were performed at a vertical 9.4 T Bruker AVANCEIII Wide Bore NMR spectrometer (Bruker; Rheinstetten, Germany) operating at frequencies of 400.21 MHz for ^1^H and 376.54 MHz for ^19^F measurements using Bruker Microimaging units (Micro 2.5) equipped with an actively shielded 40-mm gradient set (1.5 T/m maximum gradient strength, 110 µs rise time at 100% gradient switching) and ^1^H/^19^F 25-mm birdcage resonators as described previously. All MR data were acquired and analyzed by ParaVision 5.1 software.

#### *Analysis of FNP *^*19*^*F content*

^1^H rapid acquisition with relaxation enhancement (RARE): TR (repetition time) = 3500 ms, RARE factor 16, FOV = 2.56 × 2.56 cm^2^, matrix: 128 × 128, slice thickness 1 mm, scan-time 1 min. ^19^F RARE: TR = 2500 ms, TE = 3.09 ms, RARE factor 32, FOV = 2.56 × 2.56 cm^2^, matrix: 32 × 32, slice thickness 1 mm, acquisition time 5 min.

#### Cell measurements

^1^H RARE: 1 mm slice thickness, RARE factor 32, FOV = 2.56 × 2.56 cm2, matrix = 256 × 256, TR 2500 ms, TE = 4.37 ms, 1 average, scan-time 2.5 min. ^19^F RARE, 3 mm slice thickness, RARE factor 32, FOV = 2.56 × 2.56 cm^2^, matrix = 64 × 64, TR 2500 ms, TE = 4.37 ms, 128 averages, acquisition time 10 min.

#### *Matrigel/LPS *^*19*^*F measurements*

Mice were anesthetized with 1.5% isoflurane and were kept at 37 °C during the measurements. ^1^H MR reference images were recorded by multislice RARE sequence: RARE factor 16, FOV 2.56 × 2.56 mm^2^, matrix 256 × 256, slice thickness 1 mm, 5 averages, acquisition time 3 min. The corresponding ^19^F images were recorded from the same FOV using a RARE sequence with the following parameters: RARE factor 32, FOV 2.56 × 2.56 mm^2^, matrix 64 × 64, slice thickness 1 mm, 256 averages, acquisition time 21 min, TR = 2500 ms, TE = 4.37 ms. First, anatomical ^1^H scans were acquired. Subsequently, the resonator was tuned to fluorine and the corresponding ^19^F MR images covering the entire volume of the matrigel plug were recorded. For image analysis and merging of the anatomical ^1^H and ^19^F MR images, an in-house-developed software tool based on the LabView environment was used. For superimposition of the ^1^H and ^19^F MR images, the “hot iron” lookup table was applied to ^19^F datasets. To determine the total ^19^F signal in the matrigel area, regions of interest (ROI) were selected and the integral of the ^19^F signal (area × intensity) was calculated. The full protocol for in vivo MRI studies including both ^1^H and ^19^F imaging took around 30–40 min and was well tolerated by all mice which recovered from anesthesia within 5 min.

#### *Matrigel/LPS *^*19*^*F *ex vivo* measurements*

Matrigel were placed in a 500 µl reaction tube in PBS and transferred into the MRI. The matrigel was located via a ^1^H RARE sequence: RARE factor 16, FOV 2.56 × 2.56 mm^2^, matrix 256 × 256, slice thickness 1 mm, 5 averages, acquisition time 3 min. Afterwards a corresponding ^19^F scan was recorded with the same FOV.

#### Biodistribution

^1^H/^19^F measurements were performed 1, 2, 4, 8 and 24 h after injection. We applied a ^19^F FLASH sequence (FOV = 2.56 × 2.56 cm^2^, matrix 32 × 32, 5 slices, 1 mm slice thickness, flip angle = 90°, TR 50 ms, TE = 1,62 ms, 188 averages) to visualize the ^19^F content in the flowing blood. The accumulation of FNPs in liver and spleen was determined using a ^19^F RARE sequence (FOV = 2.56 × 2.56 cm^2^, matrix 64 × 64, 13 slices, 2 mm slice thickness, TR = 2500 ms, TE = 3,45 ms, RARE factor = 32, 64 averages).

### Flow cytometry

**General:** Flow cytometry was performed with a FACS Canto II (BD Biosciences, USA) or at a LSR Fortessa (BD Biosciences, USA). Cells were gated with appropriate forward/side scatter settings and thresholds for excluding debris. To omit dead cells, samples were stained with 1 µg/ml DAPI (4′,6-Diamidin-2-phenylindol, Merck). For analysis, cells were gated with FACS Diva software and the mean fluorescence intensities and/or the number of positive cells were determined, depending on the experiment.

**Murine immune cells:** The individual mouse immune cell populations were discriminated by antibody staining against CD45 (BD Biosciences, clone 30-F11), CD11b (Biolegend, clone M1/70) CD11c (Biolegend, clone N418) and Ly6G (BD Biosciences, clone 1A8); lymphocytes: CD45^+^, CD11b^−^, CD11c^−^, Ly6G^−^; classical monocytes: CD45^+^, CD11b^+^, CD11c^+^, Ly6G^−^; non-classical monocytes: CD45^+^, CD11b^+^, CD11c^−^, Ly6G^−^; neutrophil granulocytes: CD45^+^, CD11b^+^, CD11c^−^, Ly6G^+^. Cells were stained for 20 min at 4 °C, followed by washing with 200 µl MACS buffer. The different emulsions were incubated at a concentration of 10 µl/ml for the indicated period of time followed by two washing steps with 200 µl MACS buffer.

**Binding and internalization of fluorine nanoparticles:** To assess binding of FNP, ^PEG^FNP, FNP_big_ or ^PEG^FNP_big_ to murine immune cells, 1 × 10^6^ cells were incubated with 10 µl/ml of the particles over a period of 80 min at 37 °C under constant shaking in a 1.5 ml tube. At different time points 50 µl of the samples were transferred into 2 ml ice-cold MACS buffer to stop the uptake and analyzed for the rhodamine fluorescence by flow cytometry.

### Statistics

No statistical methods were used to predetermine sample size. Experiments were not randomized, and the investigators were not blinded during experiments and outcome assessment. Unless otherwise indicated, all values are given as mean ± SD. Statistical analysis was performed using OriginPro 2016 (OriginLab). Data were tested for Gaussian distribution using the D’Agostino and Pearson omnibus normality test. For comparison of parameters between the groups, a Student’s *t*-test was used.

## Supplementary Information

Below is the link to the electronic supplementary material.

Supplementary Material 1

## Data Availability

All data generated or analyzed during this study are included in this published article.
