# Supplementary material for: Impact of fluorine-containing nanoparticle PEGylation on inflammation imaging by 19F MRI
Source: Sci Rep. 2025 Nov 29;15:42937. doi: 10.1038/s41598-025-29900-8 (PMC12673124; doi:10.1038/s41598-025-29900-8)
Supplement: Supplementary file 1 — Supplementary Material 1 [file 41598_2025_29900_MOESM1_ESM.docx]

Figure S1: Long-term stability of FNPs: A) For investigation of long-term stability of the FNPs (red) and ^PEG^FNPs (grey), the size (top) as well as the PDI (bottom) were measured over 4 weeks of storage at 4 °C via dynamic light scattering. In total, we could not detect any differences for size and PDI over the complete time frame. B) For validation of the DLS measurements, we performed cryo-TEM analysis of the FNPs (red) and ^PEG^FNPs (grey). Analysis of the diameter at the beginning and 4 weeks after storage at 4 °C revealed no significant differences. C) To investigate the stability of FNPs (red) and ^PEG^FNPs (grey) at body temperature, we incubated them for 24 hrs at 37 °C in media followed by DLS measurements. We could not detect any influence on the size or the PDI due to the storage at 37 °C. Data are mean values ± SD of n = 3 independent experiments.

Figure S2: Characterization of FNP_big_: A) FNPs (red) and FNP_big_ (blue) were analyzed by dynamic light scattering regarding their size (left), size distribution (PDI) (middle) and the ζ-potential (right), indicating an increase in size and a higher PDI value as well as a lower ζ-potential for FNP_big_. B) Different cell culture lines (CHO, RAW, J774) were incubated with FNPs (red), FNP_big_ (blue) and ^PEG^FNP_big_ (grey) for 80 minutes. At different time points, the binding of the different FNPs were calculated via flow cytometric measurements. On top, histograms for the different treatments are given for the 40 min. values while below the quantification is shown. Data are mean values ± SD of n = 5 - 6 independent experiments. *** = p < 0.001 verified by student´s t-test.

Figure S3: Long-term stability of FNP_big_: A) FNP_big_ (blue) and ^PEG^FNP_big_ (grey) were analyzed by dynamic light scattering regarding their size (top) and size distribution (bottom) over 4 weeks of storage at 4 °C, indicating no change over the complete time frame. B) Additionally, cryo-TEM analysis were performed to investigate the diameter of the FNPS at the beginning and 4 weeks after storage. As indicated, we could not detect any influence on the particle size due to the storage. C) Furthermore, we investigated the stability of FNP_big_ (blue) and ^PEG^FNP_big_ (grey) after incubation at 37 °C for 24 hrs in media. DLS measurements revealed no change in size or PDI due to the incubation. Data are mean values ± SD of n = 3 independent experiments.
